# Seasonal and Geographic Variation of Southern Blue Whale Subspecies in the Indian Ocean

**DOI:** 10.1371/journal.pone.0071561

**Published:** 2013-08-13

**Authors:** Flore Samaran, Kathleen M. Stafford, Trevor A. Branch, Jason Gedamke, Jean-Yves Royer, Robert P. Dziak, Christophe Guinet

**Affiliations:** 1 Observatoire PELAGIS CNRS – UMS 3462, University of La Rochelle, La Rochelle, France; 2 Applied Physics Laboratory, University of Washington, Seattle, Washington, United States of America; 3 School of Aquatic and Fishery Sciences, University of Washington, Seattle, Washington, United States of America; 4 NOAA Ocean Acoustics Program, Office of Science and Technology National Marine Fisheries Service, Silver Spring, Maryland, United States of America; 5 Laboratoire Domaines Océaniques CNRS – UMR 6538, University of Brest, Plouzané, France; 6 NOAA Pacific Marine Environmental Laboratory, Newport, Oregon, United States of America; 7 Centre d’Etudes Biologiques de Chizé, CNRS – UPR 1934, Villiers en Bois, France; Ecole Normale Supérieure de Lyon, France

## Abstract

Understanding the seasonal movements and distribution patterns of migratory species over ocean basin scales is vital for appropriate conservation and management measures. However, assessing populations over remote regions is challenging, particularly if they are rare. Blue whales (*Balaenoptera musculus spp*) are an endangered species found in the Southern and Indian Oceans. Here two recognized subspecies of blue whales and, based on passive acoustic monitoring, four “acoustic populations” occur. Three of these are pygmy blue whale (*B.m. brevicauda*) populations while the fourth is the Antarctic blue whale (*B.m. intermedia*). Past whaling catches have dramatically reduced their numbers but recent acoustic recordings show that these oceans are still important habitat for blue whales. Presently little is known about the seasonal movements and degree of overlap of these four populations, particularly in the central Indian Ocean. We examined the geographic and seasonal occurrence of different blue whale acoustic populations using one year of passive acoustic recording from three sites located at different latitudes in the Indian Ocean. The vocalizations of the different blue whale subspecies and acoustic populations were recorded seasonally in different regions. For some call types and locations, there was spatial and temporal overlap, particularly between Antarctic and different pygmy blue whale acoustic populations. Except on the southernmost hydrophone, all three pygmy blue whale acoustic populations were found at different sites or during different seasons, which further suggests that these populations are generally geographically distinct. This unusual blue whale diversity in sub-Antarctic and sub-tropical waters indicates the importance of the area for blue whales in these former whaling grounds.

## Introduction

Prior to intensive commercial whaling, the blue whale, *Balaenoptera musculus* subspp., (Linnaeus 1758) was abundant in the Southern Hemisphere. In only a few decades, the largest living animal was hunted to the brink of biological extinction [1]–[3]. Partly in response to this overexploitation, whale sanctuaries were established in the Indian and Southern Oceans in 1979 and 1994, respectively. In these sanctuaries, two distinct subspecies of blue whales occur; the Antarctic blue whale (*B.m. intermedia*) and the smaller pygmy blue whale (*B.m. brevicauda*) [4]. The subspecies differ somewhat morphologically [5], genetically [6], and acoustically [7] and they ostensibly segregate latitudinally at least during warmer months [3]. Additionally, a separate population of pygmy blue whales is found in the northern Indian Ocean. Because this population appears to be non-migratory within a limited area between Somalia and Sri Lanka and is on a northern hemisphere breeding cycle [8], it has been proposed as a third subspecies, *B.m. indica*, by some based on the original description by Blyth (1859). Although there is little morphometric evidence for this subspecies designation, the *indica* name should have precedence over *brevicauda* if all these populations are considered to be pygmy blue whales [4], [9].

It is presumed that during the period of legal whaling in the Southern Hemisphere (1904–1964), most of the blue whales killed were the Antarctic subspecies. Pygmy blue whales were only discovered in 1959–60 [10], a few years before commercial whaling was banned by the International Whaling Commission (IWC). Antarctic blue whales were estimated to have numbered 239,000 prior to exploitation; whaling drove their populations to 0.15% of their original numbers [1], and the most recent abundance estimate is 2280 [2]. At present, although based on a limited number of sighting surveys dedicated to both subspecies, the data suggest that pygmy blue whales are not as depleted as Antarctic blue whales [3]. Both subspecies have been assessed by the IUCN’s Red List of Threatened Species [11]; Antarctic blue whales are listed as Critically Endangered, but pygmy blue whales are listed as Data Deficient due to the lack of sufficient data to assess their conservation status.

**Figure 1:**
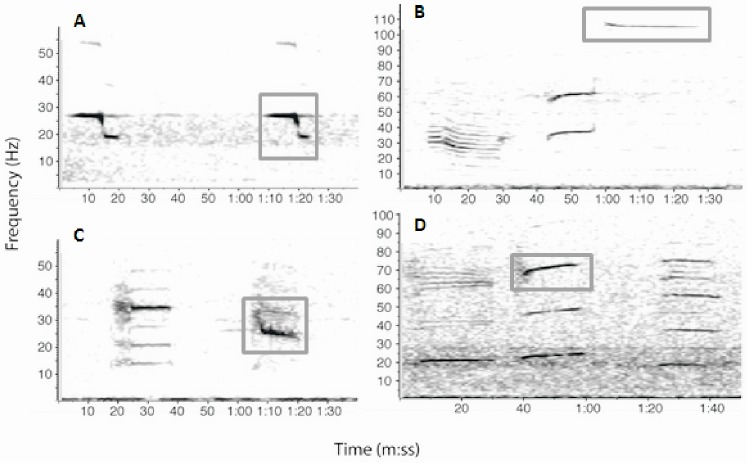
Example spectrograms from different acoustic populations of blue whale in the Indian Ocean. A: Antarctic; B: Sri Lanka; C: Madagascar; D: Australia (512 pt FFT, 87.5% overlap Hanning window). Boxes represent the part of the call used for the automated detection.

Most of our current knowledge of blue whale distribution in the Indian and Southern Oceans is derived from long-term passive acoustic monitoring. Passive acoustic monitoring has been used successfully in many oceans of the world to study the presence, migratory movements and feeding behavior of blue whales (e.g. [12], [13]). This methodology has been particularly useful in the remote sub-Antarctic and Southern Oceans [14]–[16]. Four “acoustic populations” of blue whales are found seasonally in the Indian Ocean and in the Indian Ocean sector of the Southern Ocean [16], [17]. Three of these are thought to be pygmy blue whale populations while the fourth is the Antarctic blue whale. The division of acoustic populations of pygmy blue whale subspecies is based on the time-frequency characteristics of their song signals which are readily distinguishable from one another (Figure 1, [17]). Based solely on the locations where the calls were first recorded, the four types are referred as the “Sri Lanka”, “Madagascar”, “Australia”, and “Antarctic” acoustic populations and this is the naming convention used throughout the present study. When passive acoustic monitoring, sighting and whaling data are combined, a somewhat clearer picture of the distribution of blue whales in the tropical and sub-Antarctic Indian Ocean emerges (c.f. [3]) but data gaps still exist for remote regions of the Southern and Indian Oceans.

### Antarctic Blue Whales

Antarctic blue whales are found largely south of 60°S in the austral summer where they feed, and then likely spread out over a broader range during the austral winter [3]. Although there is little evidence of wintering destinations, it was assumed that blue whales migrate between high latitude feeding and low latitude wintering areas [18]. The main feeding grounds are in the circumpolar belt between the Antarctic pack ice and the Antarctic convergence [4]. Where breeding occurs is unknown. In general, Antarctic blue whales are found at high latitudes in the Southern Ocean, close to the ice-edge during summer [3], [19]. However, based on year-round catches in South Georgia from early whaling records in 1910s and 1920s [20], [21] and, more recent year-round passive acoustic detections around Crozet Island [16], there is clearly evidence for the year-round presence of this subspecies at mid-latitudes. This suggests a wider distribution, particularly in highly productive areas of the sub-Antarctic. During autumn and winter months, some individuals may remain at high latitudes [15] while others migrate to mid- and low latitudes [16], [22].

### Pygmy Blue Whales

Pygmy blue whales are rare at latitudes south of 60°S [3],[10],[23]–[25]. Pygmy blue whales are broadly distributed between the southwestern Indian Ocean south of Madagascar to Indonesia to south-east Australia, with three populations in the northern Indian Ocean (likely the Sri Lanka acoustic population), sub-Antarctic and south-western Indian Ocean (likely the Madagascar acoustic population) and Indonesia and Australian regions (likely the Australia acoustic population; [17], [26]). The northern Indian Ocean population has been sighted and acoustically detected year-round in low northern latitudes of the Indian Ocean [9], [17], [27], although recent acoustic recordings show that at least a few individuals move south of the Equator during late summer and early autumn [16]. The sub-Antarctic and south-western population includes blue whales caught in the 1960s off Crozet, Heard and Kerguelen islands that were smaller, and which were described as the archetypal pygmy blue whale [10]. This population moves from western sub-tropical Indian Ocean waters to the sub-Antarctic during spring/summer [10], [28], [29]. Acoustic detection of pygmy blue whale calls exclusively during summer and autumn months near the Crozet Islands supports this hypothesis and highlights this region as a pygmy blue whale feeding ground [16]. The third population off Australia and Indonesia moves along the western and southern coasts of Australia in spring/summer [30], [31], before likely migrating to Indonesia in winter for breeding [3]. Recent genetic studies suggest that all pygmy blue whales seen around Australia are likely to belong to the same breeding stock [32]. Acoustic recordings also support longitudinal movements from east to west in the sub-Antarctic latitudes of the Indian Ocean [16].

Blue whales are known to concentrate in regions of high primary and secondary productivity year-round [3] and this must influence the seasonal and geographic distribution of blue whales in the Indian Ocean. In high latitudes, Antarctic krill (*Euphausia superba*) exploit the presence of abundant phytoplankton near ice edges, continental shelves and dynamic frontal regions [33] which in turn influences the seasonal presence of blue whales. Further north, sub-Antarctic and frontal areas are highly productive during austral summer [34] generating high prey densities for many marine top predators including seabirds, pinnipeds and large whales. Blue whale feeding grounds have already been reported in mid-latitudes in other ocean basins (e.g. the Southern California Bight and Monterey Bay, California, [35], [36]; Southern Chilean waters, [37]–[39]) and particularly in the southwestern [16] and south eastern [30], [31] Indian Ocean. In the northern Indian Ocean, upwelling occurs primarily during the summer (southwest) monsoon along Somalia, Oman, and India [40]–[42] but blue whales have been sighted there year-round [3], [9].

The Indian Ocean is clearly important habitat for at least four blue whale populations but little is still known about the seasonal movements and degree of overlap of these populations, particularly in the center of the Indian Ocean. In this study, we analyzed more than one year of passive acoustic recording from each of three sites located at different latitudes of the Indian Ocean basin to examine the geographic and seasonal occurrence of different blue whale acoustic populations to better understand their distribution.

## Materials and Methods

### 1 Instruments and Deployment

**Figure 2:**
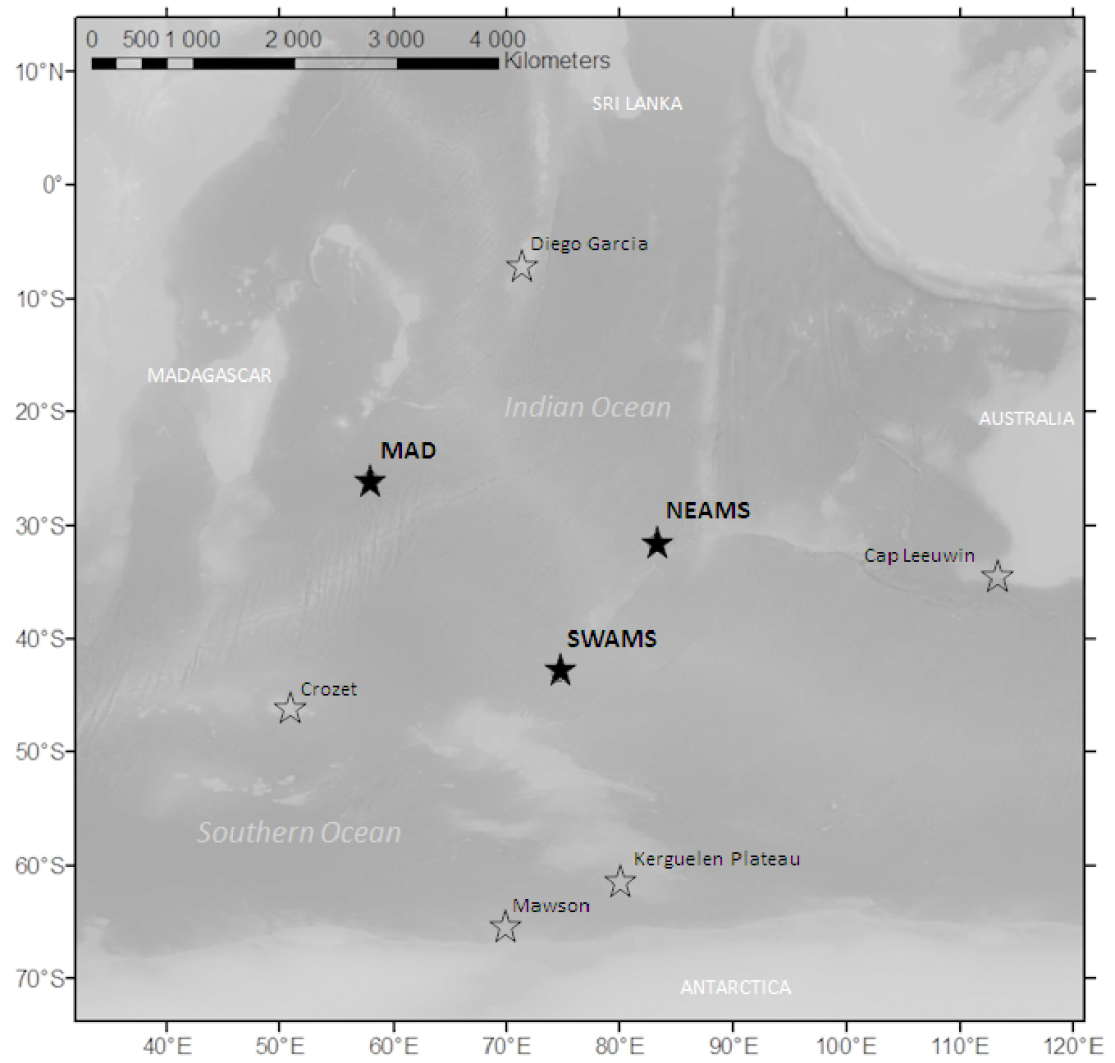
Map of the Indian Ocean. Locations of the hydrophones used in this study are shown as black stars and location of previous recordings of blue whales calls are shown as white stars.

Three autonomous hydrophone packages were deployed near the French territories in the Southern and Indian Oceans from October 2006 to January and April 2008 [43]. No specific permission was required to deploy hydrophones in the fixed location that were located in International waters. Hydrophones are passive instruments that just listen and record sounds. They have no interaction with the sea medium or the environment or animals (including endangered or protected species). The objective of the project was to monitor low-frequency acoustic signals, including those produced by large whales [44]. The three instruments were widely spaced and located in the Madagascar Basin (hereinafter referred to as MAD), about 320 nautical miles (nm) south of La Reunion Island, and 470 nm to the northeast (NEAMS) and 350 nm to the southwest (SWAMS) of Amsterdam Island (Figure 2; Table 1). Each mooring consisted of an anchor, an acoustic release, an autonomous hydrophone logging system composed of an International Transducer Corporation 1032 hydrophone, a preamplifier/filter (designed to pre-whiten ocean ambient noise spectra) and a digital recorder in a pressure-resistant titanium case and a flotation device. The mooring lines were anchored on the seafloor between 3410 and 5220 m depths and the hydrophones were deployed near the sound channel axis (SOFAR) between 1000 m and 1300 m. The instruments recorded sound continuously at a sample rate of 250 Hz (frequency range 0.1–110 Hz) and were recovered in either January 2008 or April 2008 at which time the data were downloaded for analysis.

**Table 1:** Deployment location, dates and depth for hydrophone moorings.

Station name	Location	Start date	Stop date	Instrument depth (m)
Madagascar (MAD)	26°05′S, 58°03′E	30 Oct 2006	5 Jan 2008	1300
NE Amsterdam (NEAMS)	31°35′S, 83°14′E	3 Oct 2006	25 Apr 2008	1200
SW Amsterdam (SWAMS)	42°59′S, 74°35′E	11 Oct 2006	12 Jan 2008	1000

Data analyzed were from January-December 2007.

### 2 Acoustic Data Analysis

Blue whales worldwide produce stereotyped calls with high intensity, low frequency and long duration. They also produce downswept short duration (1 to 4 s) and frequency modulated calls (90 to 25 Hz) named D calls [45], [46]. Because D calls have variable characteristics, including duration and frequency sweep, occur among feeding blue whales, and do not have obvious geographic variation (e.g. [45], [47]) this analysis used only the stereotyped blue whale calls. In order to determine the presence of blue whale calls in the acoustic recordings, automatic detection methods were used to find stereotyped signals in the recordings from October 2006 through February 2008 from all three instruments. The Antarctic blue whale call consists of three tonal units lasting ∼26 s, and repeated in patterned sequences every 40–50 s over period extending from a few minutes to hours (Figure 1a; [7], [14], [22], [46], [48]). The first component is a constant frequency tone centered on 28 Hz followed by a short frequency-modulated (FM) down-sweep from 28 Hz to 20 Hz ending with the third component, a slightly modulated tone (20–18 Hz). The Sri Lanka call consists of a three-unit phrase repeated in patterned sequences every 70–80 s over period extending from a few minutes to hours (Figure 1b; [17], [49]). The first unit begins with a long pulsive call (∼20 s) at a frequency as low as ∼20 Hz and as high as ∼45 Hz. The second unit, beginning after ∼16 s, is a FM upsweep from 56 to 72 Hz lasting ∼14 s. It is followed by a long tone (∼28 s) that sweeps from 108 to 104 Hz. The Madagascar call consists of a phrase with two long units repeated in patterned sequences every 90–100 s over a period extending from a few minutes to hours (Figure 1c; [7], [17], [48]). The first unit starts with ∼6 pulses with a center frequency of 35 Hz and a duration of 4 s followed by a 35 Hz tone lasting ∼11 s. A silence (∼30 s) separates the two-part phrase. The second unit began pulsively (2–3 pulses) with a center frequency of ∼30 Hz lasting 2–3 s followed by a FM downsweep from 30 to 22 Hz (center frequency 25 Hz) lasting ∼10 s. The Australia call consists of a phrase with two or three units repeated in patterned sequences every 200 s over periods extending from minutes to hours (Figure 1d; [17], [50], [51]). The first is a 20 Hz, 20 s duration tone that jumps to 21 Hz and continues for another 22 s; the second follows 5–10 s later and is a FM sweep beginning at 20 Hz and gradually increasing to 26 Hz during a time interval of 23 s; the last part is a near constant frequency 18–19 Hz tone that lasts 26–48 s and follows the second unit by 23 s.

Spectrogram templates consisting of units from previously described calls were searched in our records using the template detector, in the eXtensible BioAcoustic Tool package (XBAT; Bioacoustics Research Program, Cornell Laboratory of Ornithology, NY, U.S.A., www.birds.cornell.edu/brp/). The Antarctic blue whale call template consisted of one complete call. Only single units of each pygmy blue whale calls were used in those templates because they were usually above ambient noise level. We selected the third unit of the Sri Lanka call; the second unit of the Madagascar call, and the harmonic at 70 Hz of the second unit of the Australian call (Figure 1). A detection threshold was set at 0.2 to minimize false positive detections. Therefore the numbers of detections probably under-represent the presence of blue whales as faint calls or calls occurring during times of high background noise would not be detected. Detections were saved and checked for accuracy. Per month and for each call type, when the number of detected calls was under 500, each call was visually checked and when the number of detected calls over 500, a randomly chosen 10% of the detections were assessed visually to distinguish correct from incorrect detections. In order to examine geographic and seasonal variation in call detection, the number of calls per call type per month was calculated.

Although it is generally held that only male blue whales produce long repeated calls [47], the proportion of male blue whales that produce sound at any one time is not known; so, the basic assumption is that greater the number of detected calls indicates a greater number of calling whales. The seasonality of detected calls by site is based on Southern Hemisphere seasons (Summer: December–February; Autumn: March–May; Winter: June–August; Spring: September–November). The number of calls detected in each season was compared with seasonal patterns of the same call types reported in peer-reviewed and gray literature at different locations in the Indian and Southern Oceans: near Diego Garcia in the northern part of the Indian Ocean [17], [22]; near Crozet Island [16]; near Cape Leewin in the eastern part of the Indian Ocean [17], [22]; on the southern edge of the Kerguelen plateau, off Casey Station Antarctica and on the edge of the continental shelf off Prydz Bay [52].

## Results

Per month and for each call type, when the number of detected calls was under 500, false positives were manually removed from the dataset. When the number of calls was over 500, more than 94% of the double checked calls were true positives. Considering this high true positive score, detections were not corrected for detection errors to assess the seasonal variation of the number of calls detected using this automated detectors.

All four blue whale call types showed geographic and seasonal variation. Antarctic blue whale calls were the only call type recorded at all three locations and were by far the most numerous call type detected.

### 1 Antarctic Blue Whales

**Figure 3:**
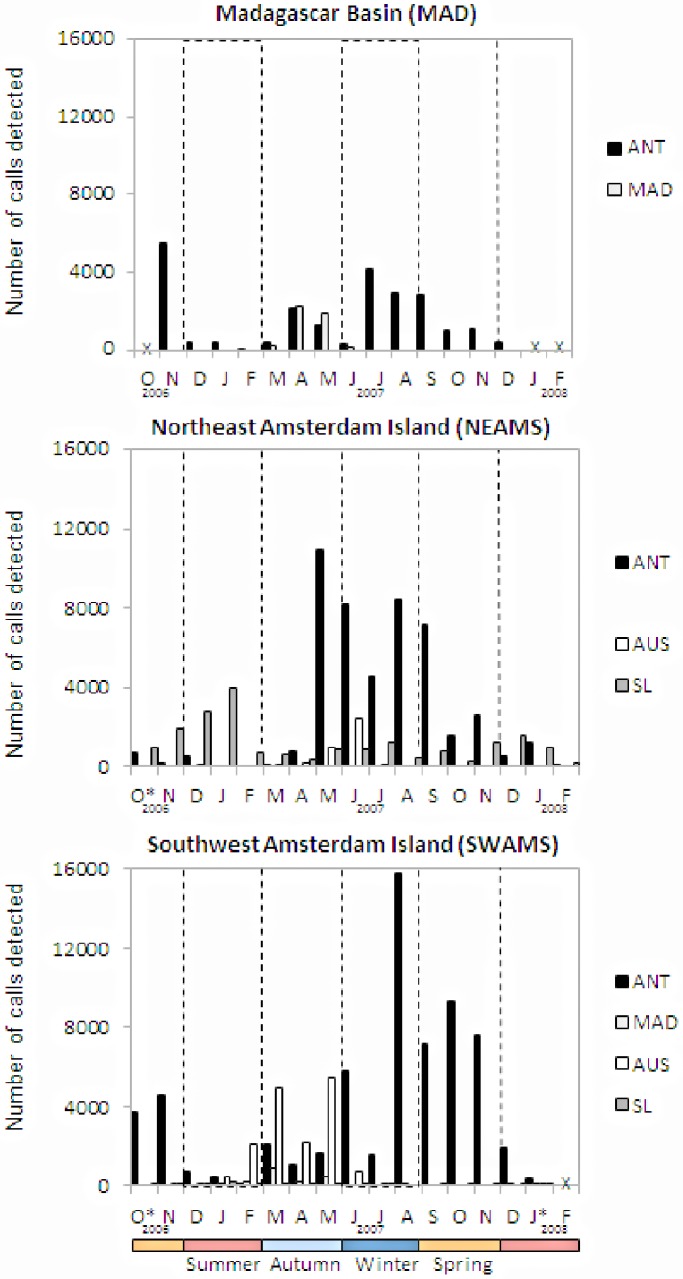
Number of blue whale calls detected at three sites. Antarctic blue whale calls (ANT - black), Madagascar calls (MAD -light grey), Australia calls (AUS - white) and Sri Lanka calls (SL – grey). X indicates no data were available and * indicates data were available only a part of the month.

**Figure 4:**
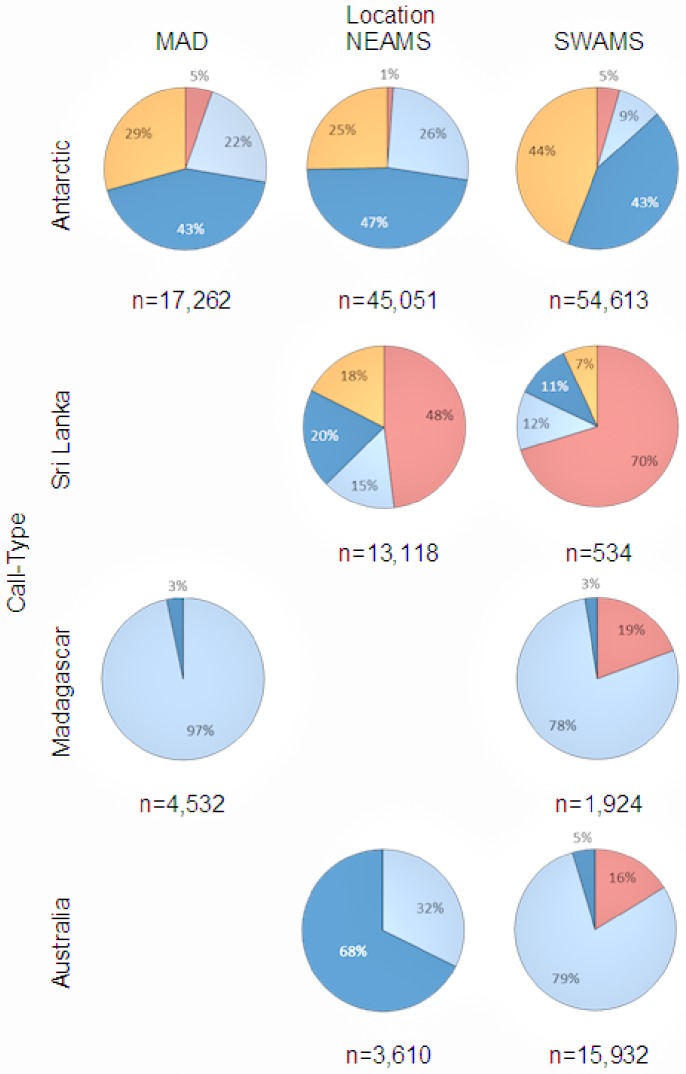
Percentage of detected blue whale calls by location, call type and Southern Hemisphere season for the year 2007 (January to December). Spring (orange), Summer (red), Autumn (light blue), Winter (dark blue).

Antarctic blue whale calls were recorded on all three stations. On both MAD and SWAMS sites, calls were recorded in all months of the year but at each station calling peaked at different times (Figure 3). At MAD call detections were the highest from July through September whereas at SWAMS the peak was from August to November. At NEAMS calls were detected from March to December (peak May-August) in 2007 and few calls were detected in January and February 2008. In 2007, three times more calls were detected at SWAMS (53,613) than at MAD (17,262) and nearly 10,000 more calls than at NEAMS (45,051). Despite differences in the total number of calls detected, the seasonal distribution of call detections was similar at MAD and NEAMS with ∼43% of all detections in winter and between 22% and 29% for both autumn and spring (Figure 4). SWAMS data displayed a similar winter percentage (∼47%), but an asymmetric distribution in the spring (44%) and autumn (9%). The percentage of summertime detections was low at all sites (5%, 1%, and 5% respectively).

### 2 Sri Lanka Call Type

Sri Lanka blue whale calls were detected year-round on NEAMS and SWAMS (Figure 3) but primarily in Austral summer (Figure 4). In 2007, very few calls were detected at SWAMS (534 versus 13, 118) and mostly during the summer, while none were detected at MAD.

### 3 Madagascar Call Type

Compared to the above call types, Madagascar calls were detected only during a limited period of the year and only at MAD and SWAMS (Figure 3 **)**. In 2007, 4,532 calls were detected at MAD from March through June, with peaks in April and May, while only half as many calls were detected at SWAMS from December to May (1,924). Madagascar calls predominantly occurred in autumn (Figure 4). No Madagascar calls were detected at NEAMS.

### 4 Australia Call Type

Records of Australian calls were limited to the eastern instruments (SWAMS and NEAMS) and for relatively short periods of the year. In 2007, four times more calls were detected at SWAMS (15,932 calls) than at NEAMS (3,610 calls), and over a longer period, January to June versus March to June, respectively (Figure 3 **)**. The distribution of call detections by season was quite different between the two sites: at NEAMS nearly 68% of calls were detected in winter while at SWAMS over 70% were detected in autumn (Figure 4). No Australian calls were recorded at the western station (MAD).

## Discussion

**Figure 5:**
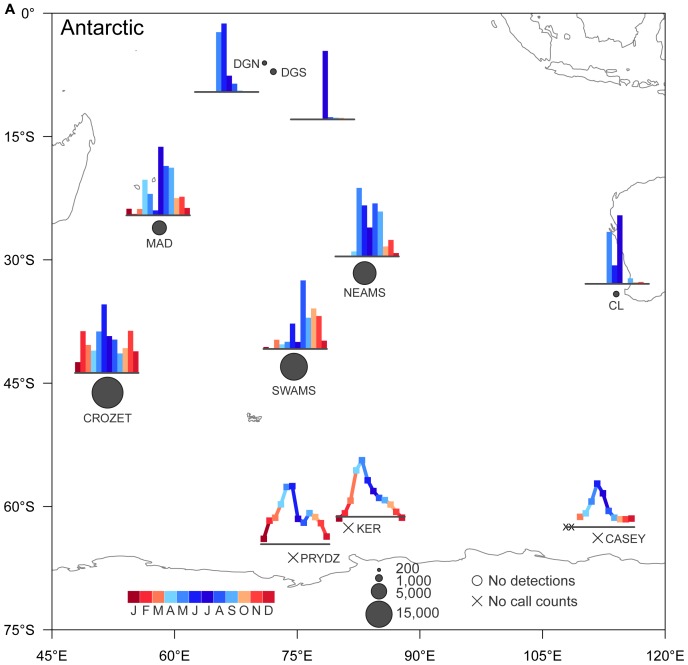
Monthly and geographic patterns of call detections of southern blue whale subspecies in the Indian Ocean. A: Antarctic call type; B: Sri Lanka call type; C: Madagascar call type; D: Australia call type. Circle size reflects the maximum number of calls received in a single month, X indicates no data were available, O indicates that no calls were detected. The bars at each location are the number of calls in that month divided by the maximum number of calls in a month at that location. The lineplots at locations PRYDZ, KER and CASEY are not number of calls detected but power spectral density ratios of acoustic energy in whale call vs. surrounding frequency bands (e.g. [14], [52]).

The vocalizations of the different blue whale subspecies and populations were recorded seasonally in three different areas of the Indian Ocean. For some call types and locations, there were spatial and temporal overlaps, particularly between Antarctic blue whale calls and the different pygmy blue whale calls. Except on the southernmost hydrophone (SWAMS), the different calls of the three pygmy blue whale acoustic populations were found at different sites or during different seasons, further suggesting that these populations of pygmy blue whales preferentially occupy different regions of the Indian Ocean, in addition to being acoustically distinct. Overall, when placed in the context of previous work (16,17), the results here show the distribution of subspecies and populations at basin scales and emphasize the importance of the Indian Ocean for blue whales (Figure 5).

### 1 Antarctic Blue Whales

Historically, Antarctic blue whale distribution has been defined by a migration pattern between high-latitude summer feeding areas and low-latitude wintering areas [19], [53]. When integrated with results from other locations, our results confirm these seasonal movements at the Indian Ocean basin scale. At all seven locations for which there are acoustic data in the Indian Ocean north of 45°S (including this study), Antarctic blue whale calls were recorded in winter months (Figure 5a). Antarctic blue whale calls were recorded at all three locations of our experiment and in almost any month of the year but the number of calls detected increased with latitude such that there were 3 times more calls at the southernmost site than at the northernmost site. Furthermore, at each site, calls were less numerous in summer and early autumn months. In summer, the decrease in the number of detected calls at all three sites suggests that Antarctic blue whales leave lower latitudes to migrate towards the Antarctic coasts. Near the Antarctic Peninsula [14] and all around the Antarctic continent [14], [15], [52], [54], Antarctic blue whale calls were detected mostly during summer and autumn months. This summer distribution of Antarctic blue whales, far from our hydrophones, may explain why the numbers of detected calls were the lowest during this period. The rest of the year, this population is dispersed in all mid- and low latitudes of the Indian Ocean, as illustrated by a similar pattern of Antarctic blue whale calls recorded on other mid- to low- latitude instruments during winter months. In the southern part of Kerguelen Plateau, the peak presence of Antarctic blue whale calls was in early April-May (Autumn) with a reduced presence through to September [52]. Off western Australia [22], in the southwestern Indian Ocean [16] and further north at Diego Garcia Atoll, these calls were recorded only in the winter [22]. The year-round presence of calling Antarctic blue whales at each location is evidence that not all individuals migrate to the ice edge, with at least some found at low and mid-latitudes during the summer.

### 2 Northern Indian Ocean Blue Whales (Sri Lanka Call Type)

Sri Lanka blue whales are presumed to be resident in the northern Indian Ocean [9], [49]. In this study, Sri Lankan calls were most often recorded on the easternmost hydrophone (NEAMS) and year-round. Most of the calls were detected during the summer months (December and January). Year-round detections at Diego Garcia were also reported but with peaks in April-June (Autumn) and November-December (Summer) [17]. No calls were detected at the western station (MAD) which suggests that this population preferentially inhabits the northeastern or central Indian Ocean basin (Figure 5b). Very few calls were detected on the southernmost hydrophone (SWAMS) and these were almost exclusively during summer. The same seasonal detection pattern was observed at similar latitudes but further west, near Crozet Island [16]. This seasonal pattern matches the seasonal movement of this subspecies in the northern Indian Ocean (Figure 5b; [9]). When the southwest monsoon dies down in about October–November, blue whales then disperse more widely (during about December to March) to other areas with seasonal high productivity. These include some parts of the southern Indian Ocean as indicated by the increase of number of detected calls at our easternmost station, especially at the beginning of austral summer. It suggests that for some individuals, feeding or breeding grounds are not limited to the equatorial and northeastern Indian Ocean.

### 3 Sub-Antarctic and South-western Indian Ocean Blue Whales (Madagascar Call Type)

Madagascar calls were only recorded in autumn and winter months at the westernmost station (MAD) and from late summer through winter months at the southernmost station (SWAMS). This seasonal pattern of Madagascar calls matches all other reports of acoustic detections in the Indian Ocean to date (Figure 5c). Madagascar calls were detected at Crozet from January to June (Summer - Autumn), and only in May and June (end of Autumn) further north near Diego Garcia [16], [17]. Moreover, visual observations of pygmy blue whales have been reported on the Madagascar Plateau during summer [29], suggesting migrations of whales between sub-Antarctic and sub-tropical waters. It is also noteworthy that a few instances of this call type were recorded near 62° S and 68°E during the summer [55]. No calls were detected at our eastern station (NEAMS) indicating that this population preferentially inhabits the western and south Indian Ocean basin. Nothing is known of where Madagascar pygmy blue whales are found later in the year (winter and spring). However, whaling reports showed catches peaked from May to September (winter) at Durban, South Africa [3]. This population may be found at the southwest of the basin, near the South African coast during winter months, but at present this is unknown. The length frequencies of blue whales caught off Durban suggest there could be some pygmy blue whales caught there in the later years. The population identity of blue whales taken at the Durban shore-based whaling station, and the current status of blue whales in western African waters deserves further study.

### 4 Indonesia and Australian Region Blue Whales (Australia Call Type)

Australia-type calls were recorded at relatively few locations in the Indian Ocean (Figure 5d). No Australia calls were detected at the western station (MAD) indicating that this population preferentially inhabits the eastern Indian Ocean basin or is generally restricted to Australia and Indonesia. Australia calls were most often recorded in autumn and at the southernmost station (SWAMS). This seasonal pattern was similar to that observed in Cape Leeuwin [17], [52] but over a longer time period than at Crozet [16]. Australia calls were also recorded later in autumn until the beginning of winter with an order of magnitude less at the easternmost hydrophone (NEAMS) which suggests that population may move to the northeast of the Indian Ocean basin during winter. Similar to the Madagascar population, Australia calls appeared only in about 6 months of the year (Figure 5d). Animals may move eastwards and northwards along coast of Australia and up to Indonesia during spring and summer as suggested by visual survey data and acoustic records [3], [51], [56].

### 5 Comparison among Populations

There were similarities in the seasonal detections of Australia and Madagascar pygmy blue whale calls, but they appeared to be geographically separated between the eastern and western Indian Ocean basins, respectively, as previously suggested (Figure 5, [17]). Both Madagascar and Australia call types were recorded with similar seasonal patterns (primarily in Autumn) at the southernmost station (SWAMS), but there was an order of magnitude more Australia calls detected than Madagascar calls. There was limited overlap of these two populations in sub-Antarctic waters in space and time.

Because we detected individual calls, rather than using an energy ratio method, which detects the acoustic energy from an unknown number of calls, it is likely that the detected calls were from animals that were relatively close to the instruments. In a comparison of the two methods on a same data set, Širovic et al (2009) [15] found that the seasonality of detection of Antarctic blue whale calls is considerably shorter for individual detections than for the detection of acoustic power in the 28 Hz band. This is likely because distant calls smear into a band between 28 Hz and 19 Hz making it impossible to distinguish (or detect) individual calls. The distance at which blue whale calls can be detected depends upon many factors including ambient noise levels, instrument type, depth of receiver and automated detection method. In the region around Crozet Island the estimated maximum detection range for both Antarctic and Madagascar pygmy blue whales is 180 km, using a single hydrophone located in the sound channel axis and an automated detector [57]. In this study, we assume that the distance at which blue whale calls can be detected was within the same range. The acoustic diversity at each location near our instruments highlights the importance of Indian Ocean at basin scales for different subspecies and populations of blue whales. This information is critical to the future conservation and management of Southern Hemisphere blue whales.

In the vicinity of the southernmost station (SWAMS), four acoustic populations of blue whales use these sub-Antarctic waters at different times of the year, but sometimes overlap in space and time. Madagascar and Australia pygmy blue whales co-occur at sub-Antarctic latitudes during the end of summer and autumn months. This is similar to the pattern reported in the southwest part of the basin [16]. The distribution range of these two acoustic populations is substantially larger than previously thought in that Madagascar pygmy blue whale calls were detected further south than previously known while Australia pygmy blue whale calls were detected further west [3], [29]–[31], [50]. Longitudinal movements might be evidence of feeding along the productive sub-Antarctic and sub-tropical fronts of the Indian Ocean [39]. These frontal areas are highly productive during austral summer [34] resulting in the presence of increased zooplankton biomass and pelagic fishes [58]. The southernmost station (SWAMS) is located between the northern and southern sub-tropical fronts of the Indian Ocean. Madagascar and Australia pygmy blue whales may be exploiting this seasonally productive area as a feeding ground before moving elsewhere in the Indian Ocean basin. Sri Lanka blue whales, which have been proposed to be resident to the northern Indian Ocean may only occasionally migrate as far south SWAMS instrument during summer months. In contrast, Antarctic blue whales seem to use sub-Antarctic waters near all three instruments throughout the year, especially during winter and spring months.

One of the biggest gaps in knowledge in blue whale acoustic ecology is a lack of understanding of the relationship between the number of calls and the number of calling whales. An increase in the number of calls from one month to another may be the result of an increase in the number of calling whales, or that calling whales stay in the detection area during a longer period of time, or that individual whales may increase their call rates seasonally. Regardless, the high number of calls highlights the importance of the sub-Antarctic Indian Ocean for Antarctic blue whales either as a wintering ground or as a final destination before moving southwards to the ice edge of the Antarctic continent.

The easternmost station (NEAMS) is roughly 1500 km to the northeast of SWAMS, at the edge of the northern sub-tropical front. Here, the detection patterns of blue whale calls are clearly different. The acoustic diversity was less since no Madagascar pygmy blue whale calls and only few Australia type calls were recorded. However, some Sri Lanka type calls were present here year-round, particularly during austral summer. This area could be a possible wintering ground for this population in addition to that hypothesized for Crozet Islands [16]. The seasonal pattern of Antarctic blue whale detections presents a clear lag when compared with the seasonal pattern at the southernmost station. Antarctic blue whales seem be present in this part of the Indian Ocean basin earlier, at the end of autumn, based on the sudden call increase observed April and May. However Antarctic blue whales leave the area earlier, at the beginning of spring, suggesting the importance of these waters for Antarctic blue whales occurs during winter months.

The northernmost station (MAD) is in sub-tropical waters 2400 km away from the southernmost station and 2500 km west of the easternmost station. The acoustic diversity here is generally poor. This area is not exploited by Australia pygmy blue whales, and perhaps the instrument is at the edge of the distribution of this population. More surprisingly, no Sri Lanka pygmy blue whale calls were detected here; this population may be distributed in a more northerly and easterly manner in the Indian Ocean. Madagascar pygmy blue whales were detected exclusively during autumn months. Unsurprisingly, fewer Antarctic blue whales were detected in these sub-tropical latitudes although the number of calls increased during winter months. During winter, Antarctic blue whales may move northwards and are widely dispersed at different latitudes in the Indian Ocean. However, the year-round presence of calls, especially in this area, far away from the main Antarctic feeding grounds, suggests that some individuals remain at lower latitudes possibly to exploit food resources present in the region. It is also possible that individuals are moving between the Antarctic and sub-Antarctic within a season such that, rather than year-round occupancy of some individuals, there is year-round occupancy of some part of a broader population. The age, reproductive status or individual condition may explain these geographic differences but there are no data to support this at present.

Blue whale sightings in the Indian Ocean are rare and difficult to obtain, and long-term passive acoustic recorders provide most of the recent information about the seasonal occurrence and movements of these animals over this remote and large area. As more information from long-term passive acoustic recorders in other regions and over longer time periods is gathered, the importance of the Indian Ocean as year-round habitat for at least four acoustic populations of blue whales, including the Critically Endangered Antarctic blue whale [11], is emphasized. Acoustic data from broadly spaced sites in the Indian Ocean illustrate the large-scale distribution of blue whales and regional differences in call types and the seasons in which they are recorded. The Crozet archipelago, for instance, appears to be a blue whale hot spot. Our understanding of the movements and distribution patterns of the different blue whale subspecies and populations can be used to direct conservation and management in the Southern Hemisphere. The unusual blue whale diversity in sub-Antarctic and sub-tropical waters shows the current importance of the area for blue whales in these former whaling grounds. In the light of the results obtained, we are confident that a unified hydrophone network would be an invaluable asset to monitor blue whale population changes at the ocean basin scale. In order better exploit these long-term acoustic datasets and eventually obtain estimates of abundance from them, information on the behavioral contexts of call production and call rates during these behaviors are needed. It is important that opportunities to obtain such data from, for example, simultaneous acoustic and visual tracking or deployment of acoustic recording tags, be pursued.
