# Correction: Seasonal and Geographic Variation of Southern Blue Whale Subspecies in the Indian Ocean

**DOI:** 10.1371/annotation/01e9ce55-8fc3-4eda-964d-755ad7e70e72

**Published:** 2013-08-21

**Authors:** Flore Samaran, Kathleen M. Stafford, Trevor A. Branch, Jason Gedamke, Jean-Yves Royer, Robert P. Dziak, Christophe Guinet

The version of Figure 5 in the article was incomplete.

The complete version of the figure is available here: http://www.plosone.org/corrections/pone.0071561.g005.tif

